# Annotations

**Published:** 1896-03-28

**Authors:** 


### Annotations.

### Dr. Jameson and South African Medicine.

South Africa, according to Dr. Laing Gordon, is almost more of a medical colony than anything else; and the conspicuous part played in its politics of recent years by Dr. Jameson is quite in keeping with its traditions from the earliest times. The first European settlement at the Cape of Good Hope was to all intents and purposes a medical settlement. As is well known, the old sailing ships, on their longer voyages, were often little better than hot-beds for scurvy. The disease, in the ships going round by the Cape to the Indies, was the cause of a vast amount of suffering and of the loss of innumerable lives. The English and the Dutch soon discovered that to put men ashore for a short time who were suffering from scurvy, and to supply them with abundance of fresh provisions, was the speediest and most economical way of curing them. Accordingly, a small station was founded by the Dutch in 1652, which had for its object the treatment of scorbutic sailors only. And that was the earliest European settlement at the Cape. From that time to the present, when a medical man, Dr. Jameson, is the second most conspicuous man in South Africa, medicine has steadily held her own there. To-day the profession seems to be established on a decidedly more satisfactory basis than is the case in Australia, or even in the mother country. Quackery, that bane of honest medicine in Great Britain, is, thanks to the energy of the South African Legislature, almost unknown. It is, indeed, so effectually suppressed that the quacks have betaken themselves, in despair, to the less harassing shelter of the Republic. There are at the present moment a good many medical practitioners in the Colonial Legislature, and one, at least, is a Cabinet Minister. There is also a "Colonial Medical Council," consisting of seven practitioners, four of whom are elected by the profession and three only are appointed by the Government. Altogether our brethren on the other side of the Equator in Africa seem to have a very good time of it indeed.

### Traumatic Neurasthenia.

The word neurasthenia stinks in the nostrils of many surgeons, and is used by them as a term of reproach, meaning something allied to shamming. That a man should appear more or less crippled by an accident, and within a few weeks of receiving substantial damages from a railway company be restored to apparent health, is a thing that many men find easier to explain by taking for granted the moral obliquity of the patient, than in any other way, and thus much injustice is sometimes done. It is well, then, to insist on the reality of the condition known as traumatic neurasthenia. Professor Victor Horsley has recently confirmed (*Clinical Journal*) what was pointed out by Charcot, viz., that in cases of traumatic neurasthenia there is generally an incubation period, during which the patient feels no ill-effects, and thinks that he has escaped without injury; and that it is only after an interval of from one to three weeks that symptoms begin, generally taking the form of pain in the back with pain down the distribution of certain superficial nerves, more especially along the distribution of the external cutaneous nerve. Such pains, although relieved by rest, are apt to return again on attempting to move even after a considerable interval of time. The patients also suffer from "exhaustion" when they make any attempt to move, or, when somewhat recovered, to walk far; they are not paralysed, but their "motor centres" soon become exhausted. Moreover, if they happen to have had any slight injury to a limb, that limb is apt to become useless, although the local injury may be obviously insufficient to cause any real paralysis. It is, in fact, a functional monoplegia, but is none the less real to the patient. Although it is rare to find true anæsthesia, sensation is often perverted; and even when the most careful examination of the eyes reveals no defect, complaint is made that the sight is worse than it used to be, and that vision is misty. Complicating all these symptoms, and, to those who are ignorant of the possibilities of this condition, throwing doubt on the *bona fides* of the case, is the fact that such patients, even when they have been genuinely injured and are in real suffering, are never reliable in their statements. This is, in fact, but another sign of the damage their nerve centres have received. They are also emotional and apprehensive, they sleep badly and have nightmare, and their volition is greatly diminished, so that although they may honestly wish to attend to their affairs they cannot do so. These symptoms are all subjective and must be investigated with due caution, but they may be none the less real, however little there may be to show for them. Everyone knows that malingering is a thing for which one must be constantly on the look-out after railway injuries, but this makes it all the more important to remember that many of the patients whose circumstances may suggest the gravest suspicion may be perfectly honest in their complaints, for traumatic neurasthenia is a real disease.

### Deaf Mutes and the School Board.

A case came before the Marylebone police magistrate among the School Board summonses last week which well illustrates the operation of that unrestricted personal freedom which is so dear to the English mind in causing degradation of the race, and shows how much further still it tends to go, and would go but for the introduction of the law, in dragging down the new generation to an even lower level than the old. The summons we refer to was one against a mother for the non-attendance of her deaf and dumb child at the school where deaf mutes are taught. The father and the mother were both deaf and dumb, as also were two of their children. Yet these people, who had carried the principle of individualism to such an extreme as to presume to beget children as imperfect as themselves, objected to their children being taught the oral system of deaf mute language, by which alone they could be restored to anything like companionship with their fellows, instead of the rude and obsolete finger language which they themselves employed. As if it were not a great enough wrong to any deaf mute offspring of deaf mute parents that it should be born at all, it seemed to be proposed to condemn it to practical isolation for all its life, just to suit the convenience of its parents during its early years. But although the law still hesitates to interfere with the multiplication of the unfit, it fortunately retains some hold over the training of such of them as are born. The magistrate advised the mother to think a little less of herself and her difficulties, and a little more of her child's welfare and best interests, and the mother ultimately promised to try to send her child to school. The law thus did at last intervene; it was, however, but another example of shutting the door after the steed was stolen.

